# Genes Associated with the Immune System Affected by Ionizing Radiation and Estrogen in an Experimental Breast Cancer Model

**DOI:** 10.3390/biology13121078

**Published:** 2024-12-20

**Authors:** Gloria M. Calaf, Debasish Roy, Lilian Jara, Carmen Romero, Leodan A. Crispin

**Affiliations:** 1 Instituto de Alta Investigación, Universidad de Tarapacá, Arica 1000000, Chile; kcrispi@gestion.uta.cl; 2 Department of Natural Sciences, Hostos College of the City University of New York, Bronx, NY 10451, USA; droy@hostos.cuny.edu; 3 Laboratorio de Genética Humana, Programa de Genética Humana, Instituto de Ciencias Biomédicas (ICBM), Facultad de Medicina, Universidad de Chile, Santiago 8380000, Chile; ljara@uchile.cl; 4 Laboratorio de Endocrinología, Facultad de Medicina, Universidad de Chile, Santiago 8380000, Chile; cromero@hcuch.cl

**Keywords:** ionizing radiation, immune responses, breast cancer, interferon, TNF, bioinformatics

## Abstract

Breast cancer is a global health issue that, when in the metastasis stage, is characterized by the lack of estrogen receptor-α, the progesterone receptor, and human epidermal growth receptor expressions. The present study analyzed the differential gene expression related to the immune system affected by ionizing radiation and estrogen in cell lines derived from an experimental breast cancer model that was previously developed; where the immortalized human breast epithelial cell line MCF-10F, a triple-negative breast cancer cell line, was exposed to low doses of high linear energy transfer α particle radiation (150 keV/μm), it subsequently grew in the presence or absence of 17β-estradiol. Results indicated that interferon-related developmental regulator 1 gene expression was affected in the estrogen-treated cell line; this interferon, as well as the Interferon-Induced Transmembrane protein 2, and the TNF alpha-induced Protein 6 gene expression levels were higher than the control in the Alpha3 cell line. Furthermore, the interferon-related developmental regulator 1, the Interferon-Induced Transmembrane protein 2, the TNF alpha-induced Protein 6, the Nuclear Factor Interleukin 3-regulated, and the Interferon-Gamma Receptor 1 showed high expression levels in the Alpha5 cell line, and the Interferon Regulatory Factor 6 was high in the Tumor2 cell line. Additionally, to further strengthen these data, publicly available datasets were analyzed. This analysis was conducted to assess the correlation between estrogen receptor alpha expression and the genes mentioned above in breast cancer patients, the differential gene expression between tumor and normal tissues, the immune infiltration level, the ER status, and the survival outcome adjusted by the clinical stage factor. It can be concluded that the genes of the interferon family and Tumor Necrosis factors can be potential therapeutic targets for breast cancer, since they are active before tumor formation as a defense of the body under radiation or estrogen effects.

## Simple Summary

Breast cancer is a disease that can be induced by high linear energy transfer alpha radiation and estrogen, leading to cell transformation and tumor formation. This study found that genes associated with immune responses, such as those of the interferon family, the TNF, and cytokines were affected by these factors. These genes, which are active before tumor formation, could be therapeutic targets for breast cancer. However, chemotherapy resistance limits treatment efficacy, necessitating further research in breast carcinogenesis to find novel biomarkers for prognosis and explore available resources.

## 1. Introduction

Breast cancer is a serious global health issue and over 70% of cases are estrogen receptor-α (ERα) positive [1,2]. However, when breast cancer cases are classified as negative for this receptor, they are highly metastatic and are characterized by the lack of the expression of ERα [3]. Since hormones such as 17β-estradiol (E) and its receptors play an important role in the pathogenesis of breast cancer, it is an important factor to be considered in the initiation of cancer.

The International Agency for Cancer Research (IARC) classified estrogens as initiators of breast cancer, as shown by the signs of carcinogenic substances [4,5]; therefore, an understanding of the role of estrogens in cancer initiation is urgently needed in the study of breast carcinogenesis. Additionally, IARC reported that ionizing radiation, a process involving the decay of uranium and thorium, produced radon, a radioactive gas that has the potential to harm cellular structures since it is a tool powerful enough to dislodge electrons through the decay of uranium [6,7]. The most significant isotopes are 222Rn and 220Rn, which are less harmful in indoor environments and not present in uranium mines. Studies have indicated that radon exposure can lead to lung cancer, including increased lung cancer risks in experimental animals that were exposed to radon [5].

Through an experimental breast cancer model developed by Calaf and Hei (2000) [8], it was demonstrated that cancer developed in a multistep fashion through exposure to environmental carcinogens such as ionizing radiation. Thus, the MCF-10F, a triple-negative breast cancer cell line [9], was exposed to low doses of high linear energy transfer (LET) radiation, such as α particles that possess a LET value similar to radon progeny particles. Such a model elucidated whether neoplastic transformation could be initiated by ionizing radiation in the presence of estrogens and whether both factors influenced the response in genes related to several processes, including motility, cell adhesion, apoptosis, oxidative stress, and so forth. In this work, it was important to determine whether genes associated with the immune system were affected by ionizing radiation and estrogen.

Tumor Necrosis Factors (TNFs) are crucial for regulating innate and adaptive immunity, which activate immunological defenses, and they are also involved in cell growth, invasion, and the metastasis of tumor cells. They are key factors for the extracellular plasma membrane, nucleus, and cytosol [10]. The stimulatory effect of TNF on cancer cells affects the anti-apoptotic cascade through the tumor promotion pathway of TNF, i.e., tumor promotion via activating NF-κB or a PKCα- and AP-1-dependent pathway of cancer cells [11]. Among the TNFs, the Tumor Necrosis Factor Alpha-induced proteins (TNFAIPs) derived from the TNF are involved in cell proliferation, invasion, and metastasis of tumor cells. The Tumor Necrosis Factor-induced protein 6 gene (*TNFAIP6*) can cause cell cycle arrest and apoptosis [10].

Resistance to chemotherapy has been linked to interferon (IFN)-target genes [12,13]. The only member of the type II interferon family that is very unique is the Interferon-Gamma (IFNγ) [14,15] that have shown autocrine signaling in breast cancer cells [16,17] through a dependent gene signature that can cause cell cycle arrest and apoptosis. The interferon-related developmental regulator 1 (*iFRD1*) gene is a transcriptional co-regulator that may be involved in controlling epithelial cell proliferation [18]. In several organs, there is an increase in *IFRD1* expression, as in human colon cancers, which predicts reduced patient survival [19]. On the other hand, the Human Interferon-Inducible Transmembrane proteins (IFITMs) correspond to a broad spectrum of antiviral factors that prevent a variety of clinically significant infections from entering the body, such as the Dengue virus, HIV-1, and influenza A virus. Among them, the Interferon-Induced Transmembrane protein 2 gene (*IFITM2*) is widely known for its antiviral properties [20].

The Interferon-Gamma Receptor 1 (*IFNGR1)* gene affects the development and metastasis of solid cancers [21,22] as demonstrated in murine mammary carcinoma 4T1 after being implanted into the mammary fat pads of IFN-gamma(−/−) mice, where the tumor developed and metastasized considerably rapidly [23].

The Interferon Regulatory Factor 6 (IRF6) is a transcription factor and a member of the Interferon Regulatory Factors (IRF) family, which controls, primarily, immune response [24]. Genes related to IRF6 have also been involved in proliferation, angiogenesis, cell adhesion, and interaction with the extracellular matrix [25]. As a member of the IRF family of proteins, it has a role in regulatory mechanisms that control mammary gland development affecting breast carcinogenesis. It has growth-inhibitory properties, and it is considered a potential tumor suppressor gene implicated in modulating the expression of the PI3K-regulatory component PIK3R2 of human breast cancer [26].

On the other hand, it is known that Interleukin-3 (IL-3) expression is limited to activated T cells, natural killer (NK) cells, and mast cell lines, whereas the transcription initiation is dependent on the activating ability of certain protein factors, such as Nuclear Factor Interleukin 3 (NFIL3), which binds to regulatory regions of the gene, typically upstream of the transcription start site [27,28].

Now, this work hypothesizes whether or not genes related to the immune system are involved in this multistep process, as depicted by a previously developed experimental breast cancer model [8], and whether such genes are present in clinical breast cancer patients. Hence, the present study aimed to analyze genes associated with the immune system such as *TNFAIP6*, *IFRD1*, *IFITM2*, *IFNGR1*, *IRF6*, and *NFIL3* in cell lines such as control MCF-10F (Ct), a triple-negative breast cancer cell line, estrogen (E), Alpha3 (A3), Alpha5 (A5), and Tumor2 (T2). All the treated cell lines were positive for estrogen receptors. This study will consider the presence of the aforementioned genes in (a) the differential gene expression derived from an Affymetrix, and (b) the presence of such genes in breast cancer patients, taking into account (i) the estrogen receptor α (*ESR1*) gene expression, (ii) gene expression of tumor and normal tissues, (iii) the estrogen receptor (ER) status, (iv) the protein levels, (v) immune cell infiltration analysis in breast cancer subtypes, and (vi) overall survival.

## 2. Materials and Methods

### 2.1. The Experimental Breast Cancer Model

**Figure 1:**
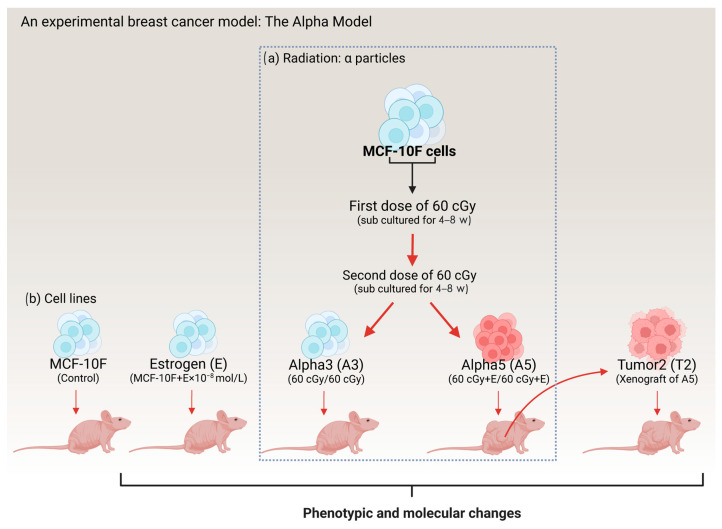
An experimental breast cancer model induced by radiation and estrogen, (**a**) MCF-10F was subjected to a single 60 cGy dose or two 60 cGy low doses of high linear energy transfer (LET) α particle radiation in the presence of 17β-estradiol or estrogen (E) and then the Alpha3, the Alpha5, and the Tumor2 cell lines were acquired. (**b**) The cell lines used in this study from the model were the parental MCF-10F (Ct) cell line, not exposed to radiation; the estrogen (E) cell line, the MCF-l0F cell line not exposed to radiation, but consistently treated with 17ß-estradiol at 10^−8^ mol/L; the Alpha3 (A3) cell line, MCF-10F exposed to double doses of 60/60 cGy α particles, a malignant but non-tumorigenic cell line; the Alpha5 cell line (A5), MCF-10F cells exposed to double doses of 60/60 cGy plus estrogen, a tumorigenic cell line; and the Tumor2 (T2) cell line, derived from the mammary tumors that developed in nude mice after being injected with the A5 cell line [8].

The model was previously described by Calaf and Hei in 2000 [8]; the model was developed with the MCF-10F (ATCC), an immortalized and negative estrogen receptor cell line. In summary, MCF-10F cells were subjected to low doses of high linear energy transfer (LET) radiation, such as α particles (150 KeV/µm, accelerated to 4 MeV using the van de Graaff accelerator) at the Radiological Research Facilities of Columbia University. The MCF-10F cell line was then sub-cultured for 12–14 weeks between doses and either a single or double dose of 30, 60, or 100 cGy of ^4^He ions was given. These distinct cell lines were cultured in an environment with or without estrogen for a period extending up to 10 months. Then, in 2013, Calaf et al. [29] used the model with the Affymetrix HG-U133A Plus 2.0 GeneChip to evaluate the process of addition as one of the processes of breast carcinogenesis in the following cell lines derived from the original model in 2000 [8] (Figure 1): (a) Ct, the parent MCF-10F cell line that was not exposed to radiation or estrogen, (b) The E cell line, MCF-l0F, consistently treated with 17β-estradiol at 1 × 10^−8^ mol/L, which did not form mammary tumors in nude mice, (c) the A3 cell line, MCF-10F, exposed to double doses of 60/60 cGy α particles, a malignant (since it formed colonies in agar) and non-tumorigenic cell line, (d) the A5, MCF-10F cells, exposed to double doses of 60/60 cGy in the presence of estrogen, a malignant and tumorigenic cell line, i.e., forming colonies in agar and being able to initiate tumors, and (e) the T2 cell line, which was derived from the mammary tumors that developed in nude mice after being injected with the A5 cell line.

### 2.2. Analysis of Microarray Gene Expression Using the Affymetrix HG-U133A Plus 2.0 GeneChip

**Figure 2:**
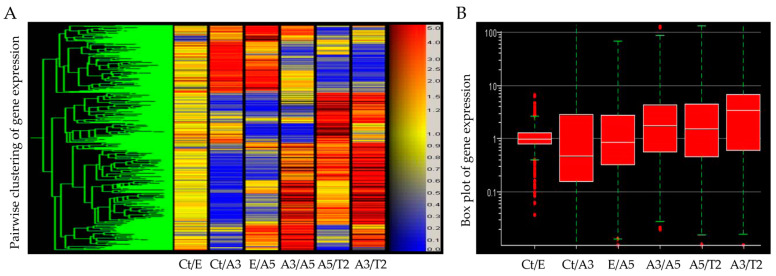
(**A**) Cluster dendrogram of gene expression and (**B**) fold-change gene expression indicated by a scatter plot of the pairwise comparative studies of the following cell lines: Ct/E (MCF-10F/estrogen); Ct/A3 (MCF-10F/Alpha3); E/A5 (estrogen/Alpha5); A3/A5 (Alpha3/Alpha5); A5/T2 (Alpha5/Tumor2), and A3/T2 (Alpha3/Tumor2). In the left panel, red denotes a higher expression, blue a lower expression, and yellow an equal expression. In the right panel, the spectrum of differential gene expression in the same paired cell line comparisons is summarized in the gene box plot, reprinted/adapted from Spandidos Publications, 2013 [29].

An Affymetrix method was used in 2013 [29] to determine which genes were affected by estrogen alone, and by ionizing radiation (Figure 2). The Affymetrix U133A oligonucleotide microarray (Affymetrix, Santa Clara, CA, USA), which comprises 14,500 genes, was used to assess gene expression in the breast cancer model, which included the cell lines Ct, E, A3, A5, and T2. The Affymetrix GeneChip Operating Software (GCOS) v1.0 ST, the Genes@Work software platform, and the discovery algorithm SPLASH (structural pattern localization analysis by sequential histograms), with a false discovery rate of 0.05, were used to quantitatively analyze arrays for gene expression.

The pairwise comparative study used the following criteria (Figure 2): (i) Ct/E compared the effect of estrogen alone versus the control to analyze the effect of estrogen; (ii) Ct/A3 compared the effect of the ionizing radiation alone versus control to analyze radiation alone; (iii) E/A5 compared the estrogen when ionizing radiation was used to assess the radiation effect; (iv) A3/A5 compared radiation alone and in combination with estrogen to analyze their combined effect; (v) A5/T2 compared both estrogen and ionizing radiation with the environment in the athymic animal; and (vi) A3/T2 assessed the role of the microenvironment and radiation alone.

### 2.3. Bioinformatic Gene Expression Analysis and Statistical Analysis

The Tumor Immune Estimation Resource v2.0 (TIMER2.0, http://timer.cistrome.org/) [30] was used to explore gene mRNA expression across breast cancer subtypes. The Gene Correlation module provided the correlation among genes, facilitated by Spearman’s test statistical analysis, and the Gene Outcome module, which employs the Cox proportional hazard model, assessed the overall survival of patients adjusted by the clinical stage factor. The Gene module delivered the association between immune infiltrates and gene expression, the statistical significance was determined by *p*-values, and partial correlation values were obtained through Spearman’s rank correlation test. The University of California, Santa Cruz (UCSC) Xena (https://xena.ucsc.edu/), accessed on 21 August 2021 [31], supplied the ER status of the genes implicated in this research, with the one-way ANOVA test calculating statistical significance. GEPIA2, Gene Expression Profiling Interactive Analysis, a web resource, delivered a comparison of the gene expression between tumor and normal tissues, represented through box plots for gene expression level distribution; the Wilcoxon test calculated the statistical significance, denoted by the number of stars (*: *p* < 0.05; **: *p* < 0.01; ***: *p* < 0.001), accessed on 18 September 2024 (http://gepia2.cancer-pku.cn/) [32]. Protein expression levels were analyzed by UALCAN, the University of Alabama at Birmingham Cancer Data Analysis Portal (http://ualcan.path.uab.edu/, accessed on 18 September 2024), including data from the Clinical Proteomic Tumor Analysis Consortium (CPTAC) [33]. HPA, the Human Protein Atlas portal, was used to carry out immunohistochemical analyses in malignant and normal tissues (https://www.proteinatlas.org/, accessed on 25 June 2024) [34]. A *p* < 0.05 was considered significant.

## 3. Results

### 3.1. Differentially Expressed Genes Affected by Ionizing Radiation and Estrogen in Normal and Transformed Breast Cell Lines

**Figure 3:**
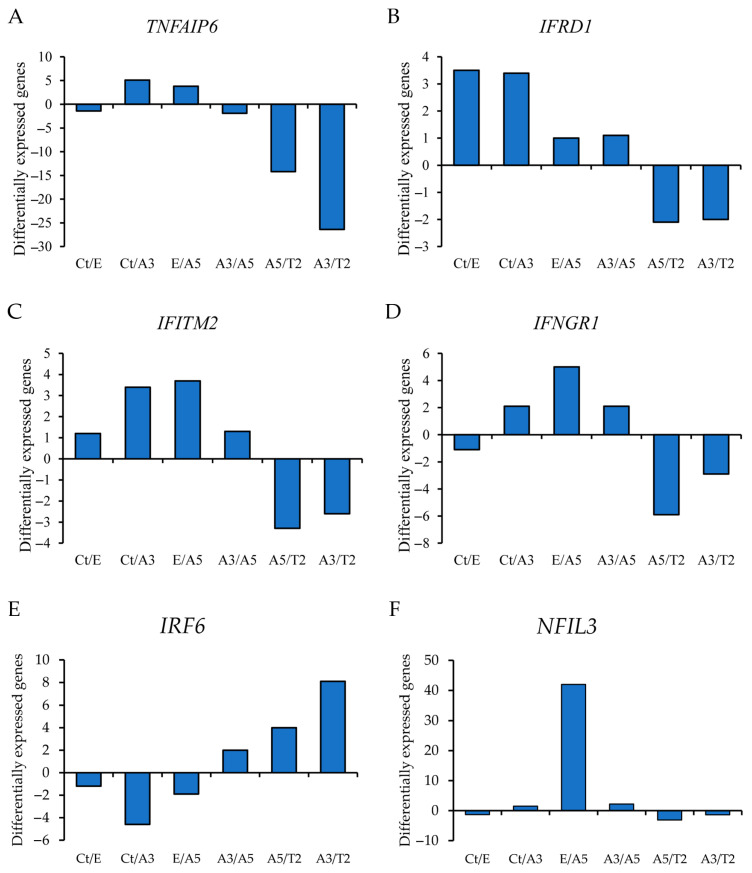
Profile of genes with differential expression acquired using an Affymetrix array (U133A) in an induced-radiation and -estrogen experimental breast cancer model, comparing genes such as (**A**) the TNF alpha-induced protein 6 gene (*TNFAIP6*), (**B**) the interferon-related developmental regulator 1 gene (*IFRD1*), (**C**) the Interferon-Induced Transmembrane protein 2 gene (*IFITM2*), (**D**) the Interferon-Gamma Receptor 1 gene (*IFNGR1*), (**E**) the Interferon Regulatory Factor 6 gene (*IRF6*), and (**F**) the Nuclear Factor Interleukin 3-regulated gene (*NFIL3*) in the following cell lines: MCF-10F/estrogen (Ct/E), Control/Alpha3 (Ct/A3), estrogen/Alpha5 (E/A5), Alpha3/Alpha5 (A3/A5), Alpha5/Tumor2 (A5/T2), and Alpha3/Tumor2 (A3/T2). The graphs were obtained from a gene expression cluster dendrogram repository from our laboratory for this article.

According to the profiling of differentially expressed genes obtained using an Affymetrix array U133A, high-LET radiation such as that emitted by radon progeny combined with estrogen caused events indicative of cell transformation and tumorigenicity in human breast epithelial cells (Figure 3). Since such events are related to alterations of genes, it was important to determine the influence of such factors in normal and transformed cell lines in these experimental conditions.

Figure 3A indicates that *TNFAIP6* gene expression was greater in the A3 and A5 cell lines than in T2. Figure 3B also shows that A3 and A5 had greater *IFRD1* gene expression than T2, whereas E and A3 were greater than the control. As shown in Figure 3C, *IFITM2* gene expression was higher in A3 and A5 than T2; A5 was higher than E, whereas A3 was higher than the control. *IFNGR1* gene expression in Figure 3D was higher in A5 than in E as well as in T2. *IRF6* gene expression (Figure 3E) was higher in T2 than in A3 and A5 and A3 lower than control. In Figure 3F, *NFIL3* was greater in the A5 cell line than in the E cell line.

### 3.2. Presence of Genes Affected by Ionizing Radiation and Estrogen: Correlation Between ESR1 Expression and Gene Under Study in Subtypes of Breast Cancer Patients

**Figure 4:**
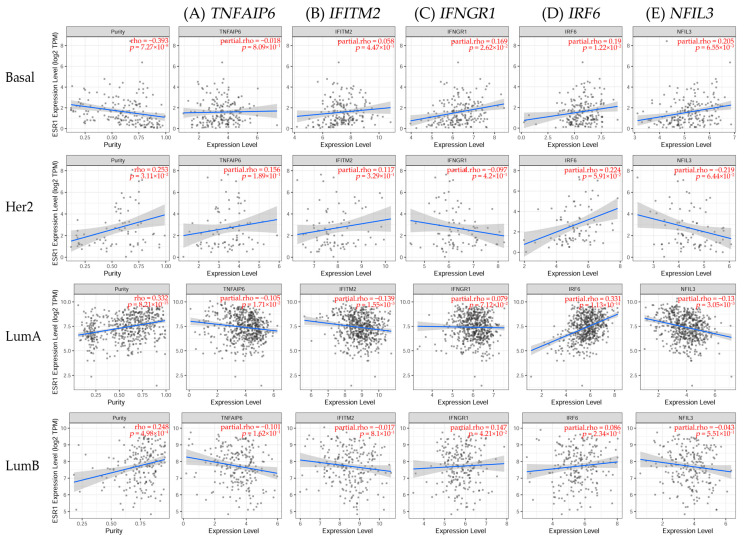
Scatter plots show the correlation between estrogen receptor alpha (*ESR1*) expression with purity adjustment (left) and (**A**) TNF alpha-induced protein 6 (*TNFAIP6*), (**B**) Interferon-Induced Transmembrane protein 2 (*IFITM2*), (**C**) Interferon-Gamma Receptor 1 (*IFNGR1*), (**D**) Interferon Regulatory Factor 6 (*IRF6*), and (**E**) Nuclear Factor Interleukin 3-regulated (*NFIL3*) gene expression levels (right) in breast cancer subtypes. The scatter plots are shown with linear regression lines. The blue lines in each plot represent the linear regression fit, which indicates the trend or relationship between the expression levels of ESR1 and the respective gene. The gray-shaded area around each blue regression line represents the confidence interval. Correlation values for each analysis are stated in red on the right (Spearman’s, *p* < 0.05). Data extracted from Tumor Immune Estimation Resource v. 2.0 (TIMER2.0), accessed on 4 January 2024 [30].

To further elucidate the regulation of differentially expressed genes affected by ionizing radiation and estrogen in transformed breast cell lines and *ESR1* gene expression, an analysis was carried out between *ESR1* and the list of genes under study. Figure 4 shows a significant (*p* < 0.05) association between *ESR1* expression with purity adjustment (left) and *TNFAIP6*, *IFITM2*, *IFNGR1*, *IRF6*, and *NFIL3* gene expression levels (right).

Results in Figure 4 indicated that there was a significant (*p* < 0.05) negative correlation between *ESR1* gene expression and *TNFAIP6* expression levels in Luminal A patients. There was also a significant (*p* < 0.05) negative correlation between *ESR1* gene expression and *IFITM2* expression levels in Luminal A cancer patients. A significant (*p* < 0.05) positive correlation between *ESR1* gene expression and *IFNGR1* expression levels was observed in Basal and Luminal B patients. A significant (*p* < 0.05) positive correlation was also observed between *ESR1* and *IRF6* gene expression levels in Basal and Luminal A breast cancer patients, and there was also a significant (*p* < 0.05) positive correlation between *ESR1* and *NFIL3* gene expression levels in Basal breast cancer patients. However, the correlation between *ESR1* and *IFRD1* was non-significant in breast cancer patients.

### 3.3. Presence of Genes Affected by Ionizing Radiation and Estrogen Associated with ER Status in Breast Cancer Patients

**Figure 5:**
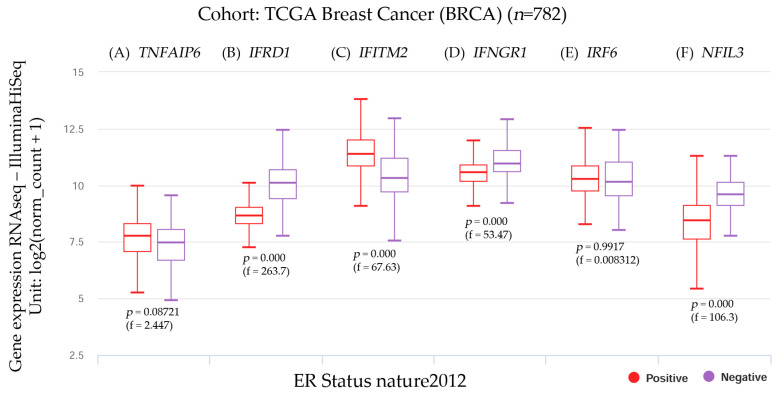
The estrogen receptor status and gene expression of (**A**) the TNF alpha-induced protein 6 gene (*TNFAIP6*), (**B**) interferon-related developmental regulator 1 gene (*IFRD1*), (**C**) Interferon-Induced Transmembrane protein 2 gene (*IFITM2*), (**D**) Interferon-Gamma Receptor 1 gene (*IFNGR1*), (**E**) Interferon Regulatory Factor 6 gene (*IRF6*), and (**F**) Nuclear Factor Interleukin 3-regulated gene (*NFIL3*) in invasive breast carcinoma. (Cohort: TCGA breast cancer (BRCA), *n* = 782) stratified by nature2012 for estrogen receptor (ER) status (one-way ANOVA, *p* < 0.05). Data retrieved from the University of California, Santa Cruz (UCSC), Xena functional genomics explorer, accessed on 4 January 2024 [31].

The ER status of *TNFAIP6*, *IFRD1*, *IFITM2*, *IFNGR1*, *IRF6*, and *NFIL3* gene expression levels is shown in Figure 5, including data from TCGA and retrieved from the Xena web database.

Results in Figure 5 indicated that there was no significant difference in gene expression and estrogen receptor status of those patients having *TNFAIP6* or *IRF6* expression levels. The median expression levels of *IFRD1*, *IFNGR1*, and *NFIL3* (10.1, 10.9, and 9.62, respectively) indicated a significant negative ER status in comparison with their counterparts (8.66, 10.6, and 8.45, respectively). However, a positive ER status was observed in those patients who had a median of 11.4 in the *IFITM2* expression level compared to its counterpart (10.3).

### 3.4. Presence of Genes Affected by Ionizing Radiation and Estrogen in Tumor and Normal Tissues Derived from Breast Cancer Patients

**Figure 6:**
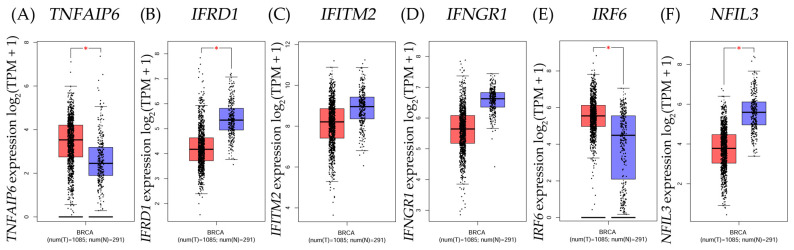
Differential gene expression levels between tumor (T) and normal (N) tissues in breast cancer (BRCA), concerning the expression of the genes under study. The box plots show the distribution of the expression levels of (**A**) the TNF alpha-induced Protein 6 (*TNFAIP6*) gene, (**B**) the interferon-related developmental regulator 1 (*IFRD1*) gene, (**C**) the Interferon-Induced Transmembrane protein 2 (*IFITM2*) gene, (**D**) the Interferon-Gamma Receptor 1 (*IFNGR1*) gene, (**E**) the Interferon Regulatory Factor 6 (*IRF6*) gene, and (**F**) the Nuclear Factor Interleukin 3-regulated (*NFIL3*) gene in tumors (red) versus normal tissues (purple) analyzed by the GEPIA2 online dataset which includes data from the TGCA repository, accessed on 18 September 2024 [32]. * *p* < 0.05.

The expression levels reported for tumor and adjacent normal tissues in invasive breast carcinoma are shown in Figure 6. Data retrieved from the Gene Expression Profiling Interactive Analysis (GEPIA2) online dataset.

Box plots in Figure 6A–F show the differential gene expression affected by ionizing radiation and estrogen in normal and tumor tissues in invasive breast carcinoma. Results show that *TNFAIP6* expression was significantly (*p* < 0.05) higher in tumor tissue than in normal tissue. *IFRD1* gene expression levels were significantly (*p* < 0.05) higher in normal tissues than in tumors. However, *IFITM2* and *IFNGR1* expression levels were non-significant. *IRF6* expression levels were (*p* < 0.05) significantly higher in the tumor tissue, whereas *NFIL3* was in normal adjacent tissues.

### 3.5. Presence of Genes Affected by Ionizing Radiation and Estrogen and Protein Expression Differences Between Tumor and Normal Tissues Derived from Breast Cancer Patients

**Figure 7:**
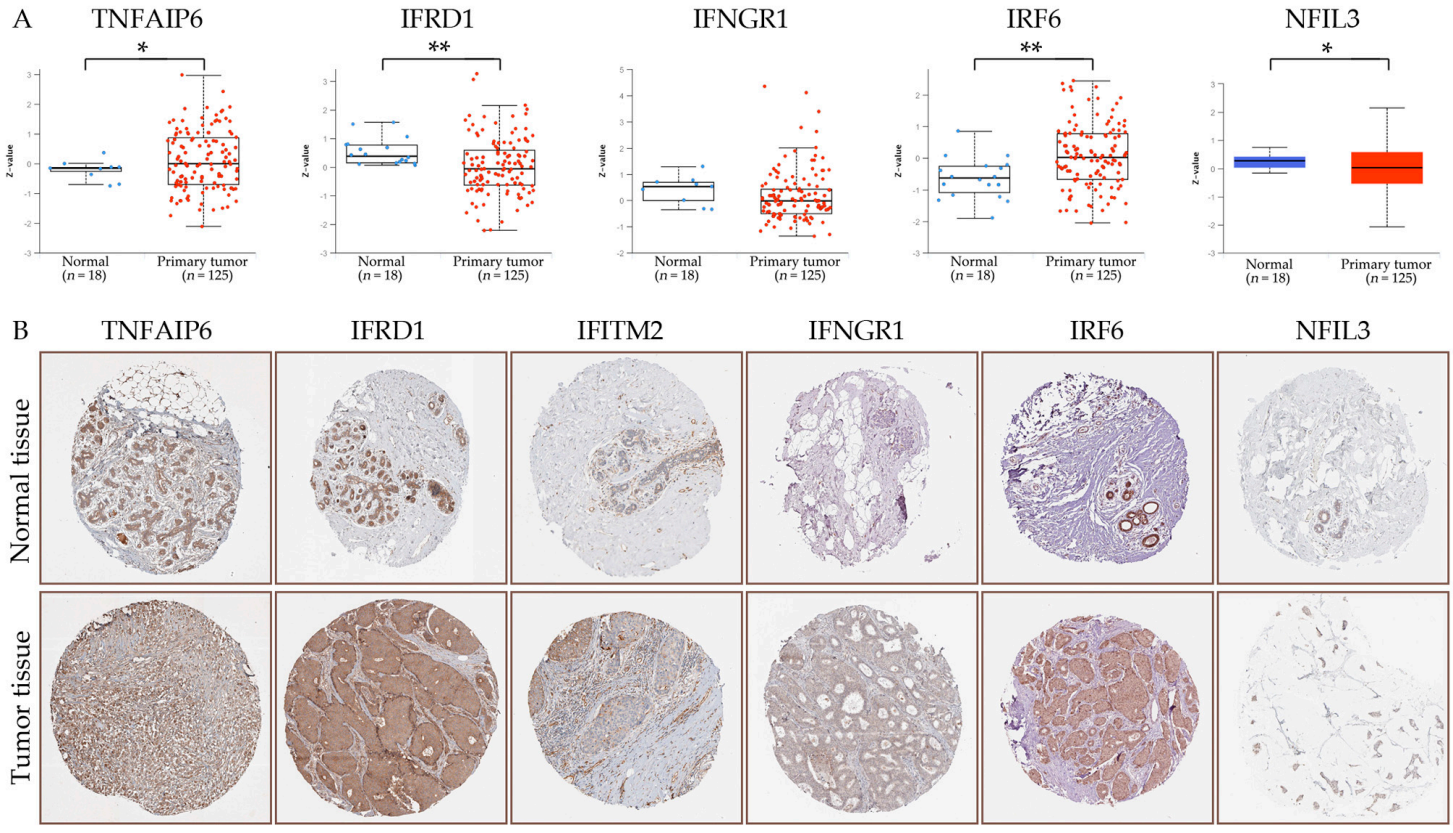
Protein expression between normal and breast cancer tissues: (**A**) Box plots depicting TNFAIP6, IFRD1, IFNGR1, IRF6, and NFIL3 protein expression levels in normal (*n* = 18) and tumor (*n* = 125) breast samples. Analysis was made using the UALCAN dataset (https://ualcan.path.uab.edu/) [33], accessed on 18 September 2024. (**B**) Immunohistochemical analysis of protein levels of TNFAIP6, IFRD1, IFITM2, IFNGR1, IRF6, and NFIL3 in invasive breast carcinoma. Samples provided by the Human Protein Atlas (HPA) repository (v23.0), available at https://www.proteinatlas.org/ [34], accessed on 25 June 2024. HPA includes data from The Cancer Genome Atlas (TCGA). * *p* < 0.05, ** *p* < 0.01.

The protein expression levels of TNFAIP6, IFRD1, IFNGR1, and IRF6 were analyzed in 125 breast cancer samples and 18 normal samples by using the UALCAN dataset. The IFITM2 and NFIL3 expression levels were not available in the dataset (Figure 7).

The analysis revealed a significant (*p* = 3.748329 × 10^−2^) difference in TNFAIP6 protein levels in tumors compared to normal samples (Figure 7A). IFRD1 was significantly (*p* = 2.276951 × 10^−3^) higher in the normal tissue than the tumor tissue, whereas IFR6 was high in tumors in comparison with normal tissues (*p* = 3.386823 × 10^−4^). NFIL3 was higher in the normal than in the tumor tissues (*p* = 4976518 × 10^–2^). IFNGR1 was non-significant and IFITM2 was not available in the UALCAN dataset. On the other hand, the HPA dataset provided an immunohistochemical analysis of TNFAIP6, IFRD1, IFITM2, IFNGR1, IRF6, and NFIL3 protein expression, which was carried out using peroxidase-conjugated antibodies such as CAB032719 for TNFAIP6, HPA024122 for IFRD1, HPA004337 for IFITM2, and HPA063871 and CAB025889 for IFNGR1 (normal and tumor tissue, respectively), HPA063121 for IRF6 and HPA003261 for NFIL3 and employing specimens derived from both normal breast tissue and invasive breast carcinoma cases.

This immunohistochemical analysis indicated that TNFAIP6, IFRD1, and IRF6 protein expression intensity was positive in normal breast tissue and ductal carcinoma. However, IFITM2 protein expression was negative in normal tissue compared to ductal carcinoma (positive). IFNGR1 and NFIL3 protein expression levels were negative in normal breast tissue as well as in ductal carcinomas.

### 3.6. Presence of Genes Affected by Ionizing Radiation and Estrogen and Immune Cell Infiltration Analysis in Breast Cancer Subtypes

Dendritic cells, macrophages, and neutrophils are the three types of innate immune cells which are very important in several processes, though the cells function in different ways depending on the biological and cellular setting; the innate immune system is involved in both healthy breast tissue and breast cancer-containing immune cells [35].

**Figure 8:**
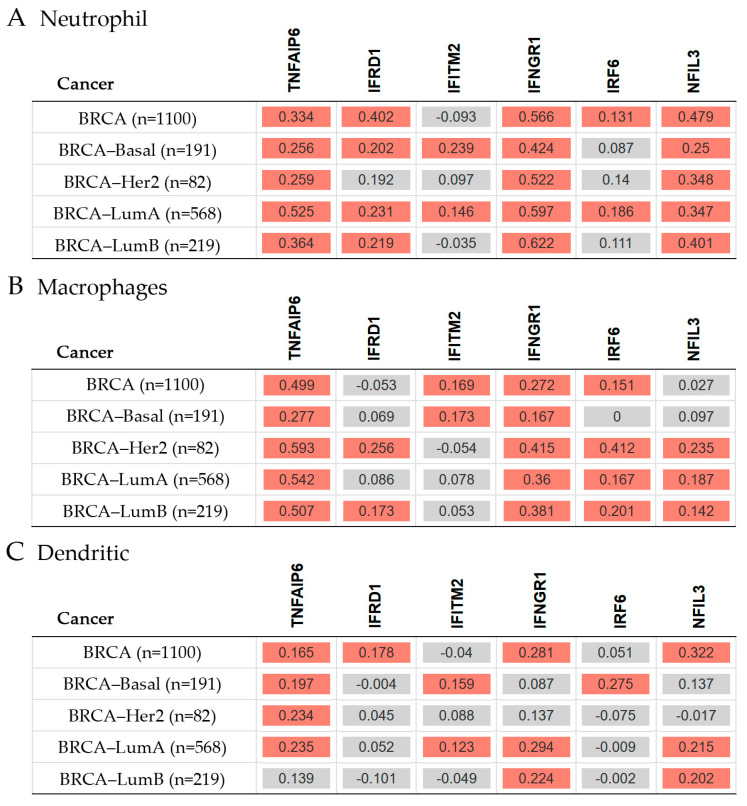
Immune cell infiltration analysis in breast cancer subtypes. The heatmap tables show the association between (**A**) Neutrophils, (**B**) Macrophages, (**C**) Dendritic cells and *TNFAIP6*, *IFRD1*, *IFITM2*, *IFNGR1*, IRF6, and *NFIL3* expression levels in breast cancer (BRCA). The red color indicates a statistically significant positive correlation (Spearman’s, *p* < 0.05) and gray denotes a nonsignificant result. Data were extracted from the Tumor Immune Estimation Resource database v. 2.0 (TIMER2.0, http://timer.cistrome.org), accessed on 4 January 2024 [30].

The heat map tables show the correlation between immune cell infiltrations and *TNFAIP6*, *IFRD1*, *IFITM2*, *IFNGR1*, IRF6, and *NFIL3* in breast cancer. To investigate the association between the genes in this study and the immune system, a correlation analysis using the TIMER2.0 dataset was conducted. The innate immune system is traditionally involved in wound healing and the removal of dead cells and cell debris [35]. Neutrophils, macrophages, and dendritic cells were the three types of innate immune cells used in this work, as seen in Figure 8.

Figure 8 shows the (A) neutrophils, (B) macrophages, and (C) dendritic cell infiltration levels. Concerning neutrophils, their association with *TNFAIP6* expression was significantly (*p* < 0.05) positive in all breast cancer subtypes, i.e., Basal, Her2, Luminal A, and Luminal B. *IFRD1* gene expression was positively correlated with neutrophils in Basal, Luminal A, and Luminal B breast cancer patients. The *IFITM2* gene expression was positively correlated with neutrophils in Basal and Luminal A patients. The correlation between *IFNGR1* gene expression levels and neutrophils was significantly (*p* < 0.05) positive in Basal, Her2, Luminal A, and Luminal B patients. The *IRF6* expression level was significantly (*p* < 0.05) positive in Luminal A patients, and the correlation between *NFIL3* gene expression levels and neutrophils was significantly (*p* < 0.05) positive in every breast cancer subtype, including Basal, Her2, Luminal A, and Luminal B.

Concerning macrophages (Figure 8B), *TNFAIP6* gene expression levels were significantly (*p* < 0.05) correlated with macrophages in Basal, Her2, Luminal A, and Luminal B cancer patients; *IFRD1* was positively correlated with macrophages in Her2 and Luminal B patients, and *IFITM2* was only correlated with macrophages in Basal patients, whereas *IFNGR1* gene expression levels were significantly (*p* < 0.05) positively correlated with macrophages in Basal, Her2, Luminal A, and Luminal B cancer patients. *IRF6* and *NFIL3* gene expression levels were significantly (*p* < 0.05) correlated with macrophages in Her2, Luminal A, and Luminal B patients.

Another correlation measurement was carried out by analyzing dendritic cells, whereby it was seen that *TNFAIP6* correlated positively with dendritic cells in Basal, Her2, and Luminal A patients with a significant (*p* < 0.05) positive difference. *IFRD1* was not significant concerning the infiltration of dendritic cells. The correlation between *IFITM2* expression and dendritic cell infiltration was significantly (*p* < 0.05) positive in breast cancer Basal and Luminal A patients, whereas both *IFNGR1* and *NFIL3* expression levels had a significant (*p* < 0.05) positive correlation with dendritic cells in Luminal A and Luminal B patients, and *IRF6* was only correlated with dendritic cells in Basal cancer patients.

### 3.7. Presence of Genes Affected by Ionizing Radiation and Estrogen and Overall Survival in Breast Cancer Patients

**Figure 9:**
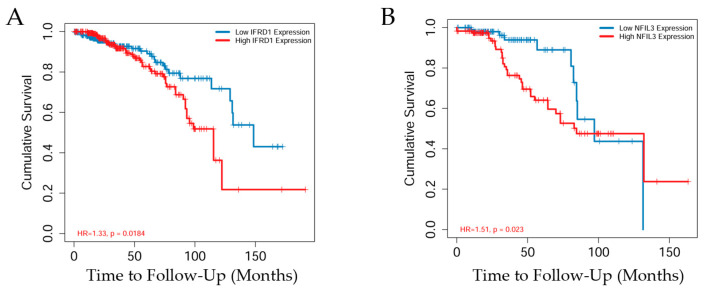
The Kaplan–Meier curve graphs show (**A**) *IFRD1* and (**B**) *NFIL3* expression levels in breast cancer patients. Patients were grouped in high (red line) and low (blue line) *IFRD1* and *NFIL3* expression categories. Analysis performed with TIMER2.0 [30] and adjusted by clinical stage factor, accessed on 4 January 2024.

The prognosis for breast cancer patients was based on the outcome of gene expression adjusted by the clinical stage factor. Patients were categorized according to the gene expression level, i.e., high expression and low expression. Figure 9 shows the survival of breast cancer patients analyzed by TIMER2.0 and determined by the Cox proportional hazard model, which evaluated the outcome significance of gene expression adjusted by the clinical stage factor.

Overall survival analysis showed that *TNFAIP6*, *IFITM2*, *IFNGR1*, and *IRF6* expression levels were not significant, whereas *IFRD1* (*n* = 568) indicated a significantly (*p* < 0.05) increased risk in Luminal A breast cancer patients, along with *NFIL3* (*n* = 219) in Luminal B patients with similar significance. The Kaplan–Meier curve shows that patients with high *IFRD1* expression exhibited an approximate 0.2 value on the cumulative survival axis at 150 months; this group displayed a higher hazard ratio (HR = 1.33), expressing a higher risk of mortality in comparison with the low *IFRD1* group (Figure 9A). Patients with low *IFRD1* expression showed an approximate value of 0.5 on the cumulative survival axis at 150 months. Patients with high *NFIL3* gene expression showed a value of 0.25 on the cumulative axis at 150 months, whereas Luminal B patients with low *NFIL3* expression exhibited zero cumulative survival value at 130 months (HR = 1.51) (data retrieved from TIMER2.0).

## 4. Discussion

The present work summarizes those genes related to the immune system such as those from the protein family known as Tumor Necrosis Factors (TNFs) that are involved in cell proliferation, invasion, metastasis and apoptosis, such as TNFAIP6, which is active at earlier stages of cell transformation, and also those genes that are potential indicators of the response of breast carcinogenesis to chemotherapy, such as the interleukins. Resistance to chemotherapy has also been linked to interferon-target genes that can cause cell cycle arrest and apoptosis. Among them is the interferon-related developmental regulator 1 (IGRD1) protein that plays a role in regulating the process of inflammation.

*TNFAIP6* gene expression was shown to be greater in the A3 and A5 cell lines than a T2 cell line derived from a tumor in the athymic mice after injection of A5 cell line, indicating that Tumor Necrosis Factor is crucial for regulating innate and adaptive immunity by activating immunological defenses even before the tumor is formed by the effects of radiation and estrogen. Then, the presence of these genes was analyzed in breast cancer patients. Such analysis indicated that the *TNFAIP6* gene expression was higher in the tumor than in the normal adjacent tissues. However, the immunohistochemical analysis indicated that TNFAIP6 protein expression intensity was positive in both normal breast tissue and ductal carcinoma. There was a negative correlation between *TNFAIP6* and *ESR1* gene expression, specifically in Luminal A, as well as no significant difference concerning ER status. On the other hand, a positive correlation was found between *TNFAIP6* gene expression and neutrophil and macrophage infiltration in all breast cancer subtypes, including Basal, Her2, Luminal A, and Luminal B, as well as with dendritic infiltration except in the case Luminal B patients. Such a reaction probably helped the defense of the body resulting in no increased risk in the survival of such patients.

*IFRD1* gene expression was found to be greater in A3 and A5 than in the T2 cell lines, and E cell line had higher *IFRD1* gene expression than the control, demonstrating that either radiation or estrogen alone, or radiation combined with estrogen, was activated in the early stages of breast carcinogenesis, but not in the tumor. On the other hand, *IFRD1* gene expression was higher in normal tissues than tumor tissues of breast cancer patients, indicating that the role of the immune system is present in any stage of carcinogenesis. The immunohistochemical analysis indicated that IFRD1 protein expression intensity was higher in normal breast tissue than in ductal carcinoma. There was a non-significant difference in the correlation between *IFRD1* and *ESR1* gene expression levels in breast cancer patients; additionally, those patients had significantly negative ER statuses, corroborating the lack of a relation between its action and the hormonal status of the patient. Furthermore, there was a positive correlation between *IFRD1* gene expression and neutrophil infiltration in Basal, Luminal A, and Luminal B cancer patients and between *IFRD1* gene expression and macrophage infiltration in Her2 and Luminal B patients. However, there was no significant association between dendritic cells and breast cancer subtypes, indicating the important role of the immune system in certain types of breast cancer subtypes. *IFRD1* gene expression levels indicated increased risk in the outcome of Luminal A breast cancer patients, showing a role in the prognostic value for patient survival.

*IFITM2* gene expression was greater in the A3 cell line than the T2 cell line, probably due to radiation, as well as in the A5 over the E cell line, also due to radiation action. A non-significant difference in *IFITM2* expression between the tumor tissue and normal adjacent tissues was observed, which was correlated with immunochemical studies that showed negative in normal tissue compared to ductal carcinoma, where it was positive. In addition, a negative correlation between *IFITM2* and *ESR1* gene expression in the Luminal A subtype of cancer patients was seen, indicating that ER expression is not important for interferon action; however, there was a positive correlation between *IFITM2* gene expression and positive ER status in breast cancer patients, showing the importance of ER status as a complementary marker for the immune system for future treatment. Additionally, *IFITM2* gene expression was positively correlated with neutrophil infiltration in Basal and Luminal A patients, and between macrophage infiltration and *IFITM2* in Basal patients. There was a positive correlation between dendritic infiltration and *IFITM2* expression in Basal and Luminal A patients. All these results indicated that IFNs were present under the effect of important infiltrations affecting the tissues before tumor formation. A non-significant difference was observed between *IFITM2* gene expression and the clinical outcome concerning the relevance of immunological genes across breast cancer subtypes. The authors of [36] demonstrated that IFITMs played a role in regulating immunological responses, which included adaptive T-cell and B-cell responses and innate antiviral and inflammatory responses, which could serve as a diagnostic tool for aggressive and resistant treatment in cancers with pathogenic functions of their own.

*IFNGR1* gene expression was higher in the A5 than in E and T2 cell lines, indicating the effect of radiation and estrogen combined on *IFNGR1* gene expression before tumor formation. On the other hand, *IFNGR1* gene expression was not significantly different between normal tissues and tumors, indicating that the IFNs could act before tumor formation. The immunohistochemical analysis indicated that IFNGR1 protein expression was negative in normal breast tissue as well as in ductal carcinomas. There was also a positive correlation between *ESR1* expression and *IFNGR1* expression levels in Basal and Luminal B patients, indicating that ER gene expression was important for interferon action. However, *IFNGR1* gene expression had a negative ER status indicating a lack of significance between such status and the immunological system. On the other hand, a positive correlation was found between *IFNGR1* gene expression and neutrophil infiltration in all breast cancer subtypes, i.e., Basal, Her2, Luminal A, and Luminal B breast cancer; similarly, the correlation between macrophage infiltration and *IFNGR1* gene expression was positive in every breast cancer subtype. However, it had a significant positive correlation with dendritic infiltration only in Luminal A and Luminal B cancer patients. There was a non-significantly increased risk to survival in patients exhibiting *IFNGR1* gene expression.

*IRF6* gene expression was higher in the T2 cell line than A3 cell line, demonstrating the importance of the environment proper of the tumor, including radiation and estrogen effects. In addition, IR6 gene expression was higher in tumors than in normal tissues, corroborating previous such results. A significant positive correlation between *IRF6* and *ESR1* expression was found in Basal and Luminal A subtypes, giving a good marker for breast cancer patients. However, there was no significant difference between *IRF6* gene expression and ER status, indicating that this is not a good marker for therapeutic reasons, since treatment with estrogen is indicated through this kind of result. The immunohistochemical analysis indicated that IRF6 protein expression was positive in normal breast tissue and ductal carcinoma. Furthermore, the *IRF6* expression level was positively correlated with neutrophil infiltration in Luminal A; with macrophages in Her2, Luminal A, and Luminal B patients; and with dendritic infiltration in the case of Basal-subtype indication of the immunological response. There was no significant difference in those patients with *IRF6* gene expression concerning the risk of survival, and therefore, there was no effect on survival.

The *IRF6* also played a role as a regulator of the cell cycle and transformation affecting Notch signaling in MCF-10A cells and stem cell control by determining luminal cell fate in a normal breast cell line [37]. Authors have indicated that IRF6 and maspin together control the cell cycle and differentiation of mammary epithelial cells [38]. Others [39] showed that IRF6 directly interacted with the tumor suppressor maspin in the 1436N1 immortalized normal mammary epithelial cell line. It was found that IRF6 was accumulated due to cell cycle arrest in the non-tumorigenic immortalized breast epithelial cell line MCF-10A, but ectopic expression by adenoviral vectors resulted in reduced cell counts in breast cancer cell lines such as the MCF7 and MDA-MB-231 cell lines [40]. Another study found that the overproduction of ErbB2 by breast epithelial cells induced down-regulation of *IRF6* [41].

*NFIL3* gene expression was greater in the A5 cell line than the E cell line, indicating that radiation influenced such effects. On the other hand, NF was higher in normal tissue than tumor tissue, and the immunohistochemical analysis indicated NFIL3 protein expression was negative in normal breast tissue as well as in ductal carcinomas. There was a significant positive correlation between *NFIL3* and *ESR1* expression in Basal subtype patients; on the other hand, there was a significant negative correlation between *NFIL3* and *ESR1* gene expression in Luminal A subtypes. There was also a significantly negative ER status. These are interesting findings, since other authors have shown that *NFIL3* increased tumor progression of triple-negative breast cancer type by suppressing NFKB inhibitor alpha *(NFKBIA)* transcription [42].

**Scheme 1:**
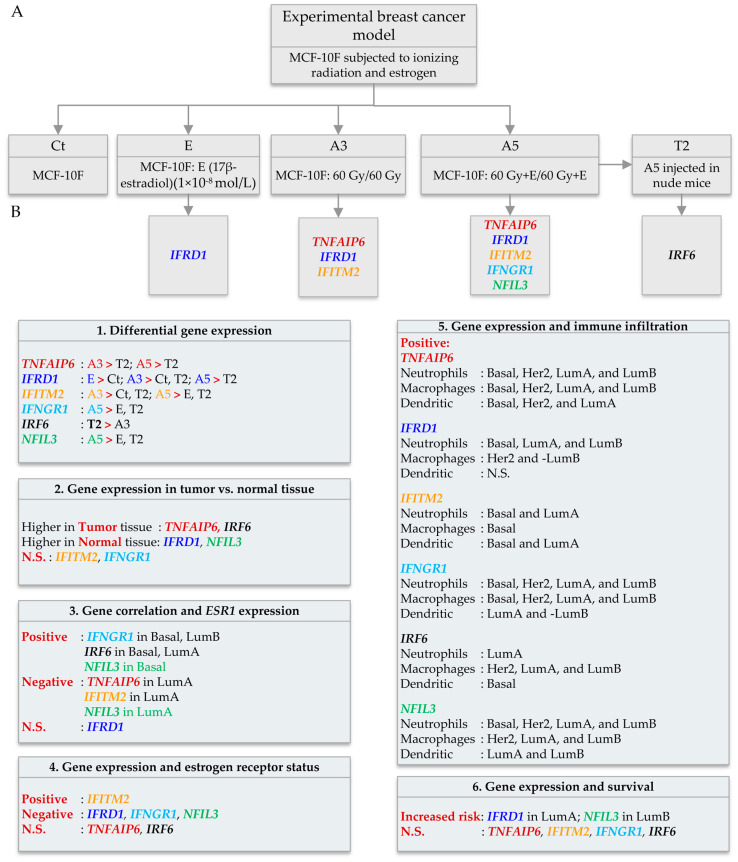
Summary of the following: (**A**) The experimental radiation and estrogen breast cancer model. (**B**) The presence of genes under study in several cell lines from the model. (**1**) Differential expression of genes analyzed by Affymetrix in several cell lines from the model. Summary of data retrieved from different online datasets. (**2**) Gene expression in tumors versus normal adjacent tissues. (**3**) Correlation between genes under study and estrogen receptor alpha (*ESR1*) expression level. (**4**) Gene expression and estrogen receptor (ER) status. (**5**) Gene expression and immune infiltration in breast cancer subtypes. (**6**) Overall survival analysis of selected genes. The colors are only used as a visual aid. Abbreviations: Ct: Control; E: estrogen cell line; A3: Alpha3 cell line; A5: Alpha5 cell line; T2: Tumor2 cell line; N.S.: Non-significant; LumA: Luminal A; LumB: Luminal B.

Furthermore, there was a positive correlation between *NFIL3* gene expression and neutrophil infiltration in every cancer subtype, including Basal, Her2, Luminal A, and Luminal B. Additionally, *NFIL3* gene expression levels were positively correlated with macrophages in Her2, Luminal A, and Luminal B patients with similar significance. Results also showed that *NFIL3* positively correlated with dendritic infiltration in Luminal A and Luminal B cancer patients, indicating the importance of the immunological system in the defense of the body. Concerning overall survival in patients with *NFIL3* expression, there was an increased risk in the outcome of Luminal B breast cancer patients that were positive for ERα. It is interesting to mention that A3 and A5 cell lines are also positive for ERα in comparison with normal immortalized MCF-10F, which is negative for such a receptor. The A3 and A5 cell lines are not tumor cell lines, but they are malignant and tumorigenic, respectively, indicating that *NFIL3* could be a potential immunological and prognostic biomarker for breast cancer. The expression and functional significance of *NFIL3* have been associated with ovarian cancer and could also be a potential biomarker for this type of cancer [43]. When hypoxia-related risk factors and clinical relevance were studied in breast cancer, NFIL3 risk scores were identified as independent prognostic indicators for breast cancer patients [44]. A summary of these findings can be seen in Scheme 1.

In general, *TNFAIP6*, *IFRD1*, *IFITM2*, *IFNGR1*, and *NFIL3* would be good markers for early diagnosis, since they are all present before the tumor formation in the model; however, *IRF6* could be a good marker for tumor development in the patient, which could help to predict the response of the immune system in breast cancer patients. On the other hand, *IFNGR1*, *IRF6*, and *NFIL3* were present in the Basal subtype as well as before tumor formation in the cell line of the model, indicating that they could be good markers for Basal subtype breast cancer patients. Additionally, *IFNGR1* could also be used as a marker for Luminal B and *IRF6* for Luminal A patients.

It is important also to point out that neither estrogen nor radiation alone formed tumors in the nude mice, whereas the combination provided the tumor cell lines. Even though the exposure of the cell lines both to radiation and estrogen is not relevant in a real-life context, it is important to understand the defense mechanisms of the immune system regarding those cells when exposed to either of those two factors.

This work was primarily based on the use low doses of ionizing radiation, similar to radon, which is not comparable to the gamma radiation used in clinic, since our aim was to determine the effect of environmental substances and estrogen on breast cancer initiation. Therefore, the association of the cell lines derived from the model and the genes under study can only suggest the possible influence of such factors on gene expression which could indicate how the immune system is affected by ionizing radiation and estrogen in an experimental breast cancer model. Further studies are being considered to determine the influence of ionizing radiation and estrogen on the phenotypic properties of breast cancer subtypes and the molecular mechanisms of action involved in the processes of initiation.

## 5. Conclusions

This work revealed that several genes associated with immune responses were affected by radiation or estrogen or by their combination in the experimental breast cancer model. These studies concluded that genes belonging to the interferon family, the TNF, and cytokines have the potential to be therapeutic targets for breast cancer since they are active before tumor formation in the defense of the body under poor conditions after radiation or estrogen effects. Extensive research into the treatment of breast cancer is urgently needed; however, chemotherapy resistance is an important issue that limits the efficacy of all efforts for its treatment. Therefore, new research in the field of breast carcinogenesis will aid in the search for novel biomarkers to predict prognoses and consider new available resources.

## Data Availability

TIMER2.0 is freely available at http://timer.cistrome.org (accessed on 6 August 2021), reference number [30]; UCSC Xena online exploration tools are freely available at http://xena.ucsc.edu/ (accessed on 20 August 2021), reference number [31]. The Gene Expression Profiling Interactive Analysis (GEPIA2) is freely available at http://gepia2.cancer-pku.cn/ (accessed on 18 September 2024), reference number [32]. The University of Alabama at Birmingham Cancer Data Analysis Portal (UALCAN) is available at https://ualcan.path.uab.edu/ (accessed on 18 September 2024), reference number [33]. The Human Protein Atlas (HPA), v23.0, is freely available from https://www.proteinatlas.org/ (accessed on 25 June 2024), Reference number [34]. The data generated in the present study may be requested from the corresponding author.
